# Supplementary material for: Coenzyme A corrects pathological defects in human neurons of PANK2‐associated neurodegeneration
Source: EMBO Mol Med. 2016 Aug 11;8(10):1197–211. doi: 10.15252/emmm.201606391 (PMC5048368; doi:10.15252/emmm.201606391)
Supplement: Supplementary file 1 — Expanded View Figures PDF [file EMMM-8-1197-s001.pdf]

Expanded View Figures

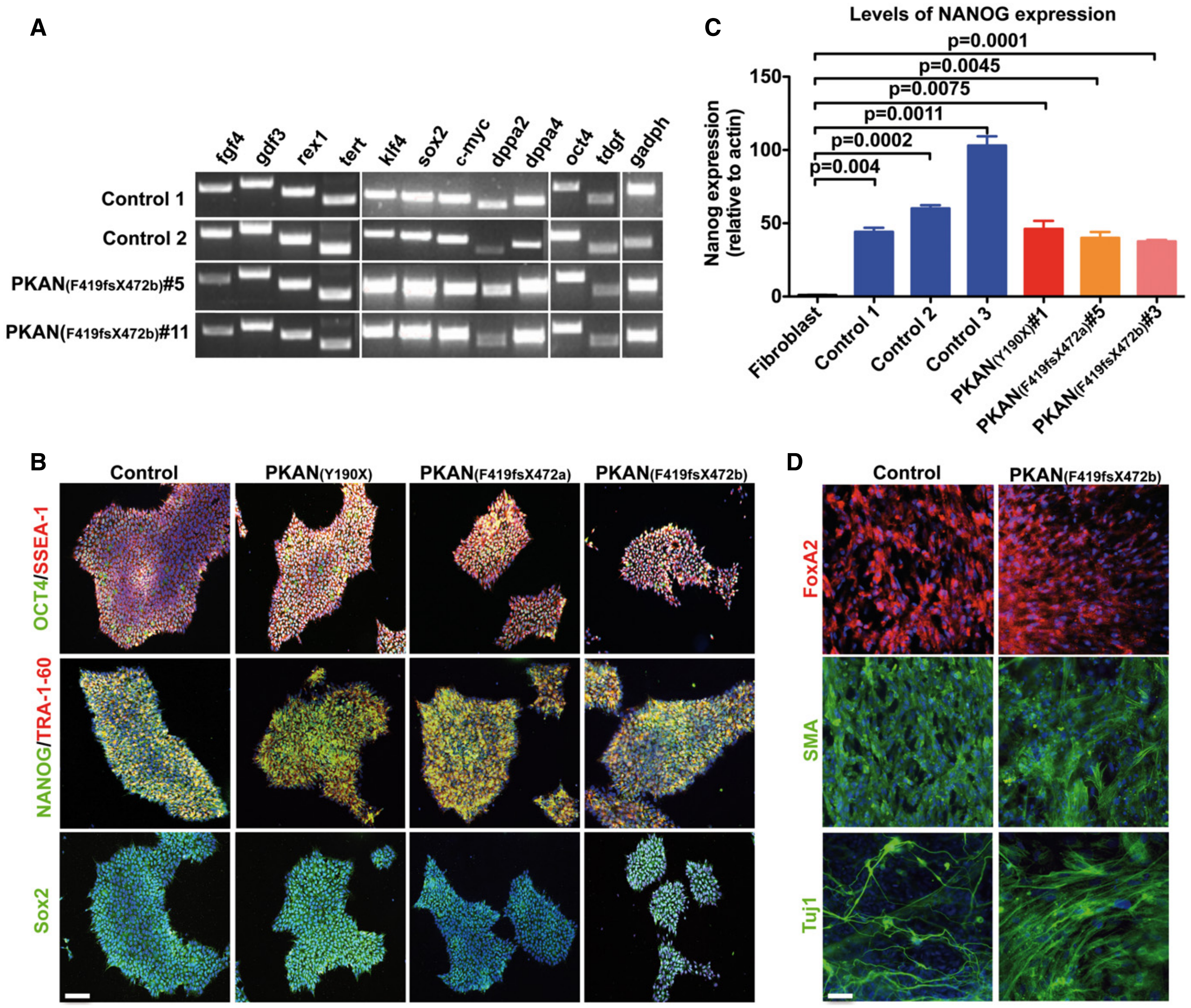

Figure EV1. Characterization of hiPSC clones obtained from fibroblasts of controls and PKAN patients.

A RT–PCR analysis of pluripotency markers expressed by hiPSC clones. Fgf4: fibroblast growth factor 4; Gdf3: growth differentiation factor-3; Rex1: reduced expression 1; Tert: telomerase reverse transcriptase; Klf4: Kruppel-like factor 4; Sox2: sex determining region Y-box 2; c-myc: Myc proto-oncogene protein; Dppa2/4: developmental pluripotency-associated 2/4; Oct4: octamer-binding transcription factor 4; Tdgf: teratocarcinoma-derived growth factor; Gapdh: glyceraldehyde 3-phosphate dehydrogenase.

B Representative images of hiPSC colonies immunostained with pluripotency markers: Oct4, SSEA-1, NANOG, Tra-1-60, Sox2. Nuclei stained with Hoechst. Scale bar 100 μm.

C qRT–PCR of NANOG expression levels in hiPSCs. All clones expressed significantly higher levels of NANOG than control fibroblasts. Data presented as means + SEM of three independent replicates (unpaired two-tailed *t*-test).

D Representative images of hiPSCs differentiated *in vitro* into all three germ layers (endoderm, FoxA2; mesoderm, SMA; ectoderm, Tuj1). Nuclei were stained with Hoechst dye. Scale bar 20 μm.

 © 2016 The Authors

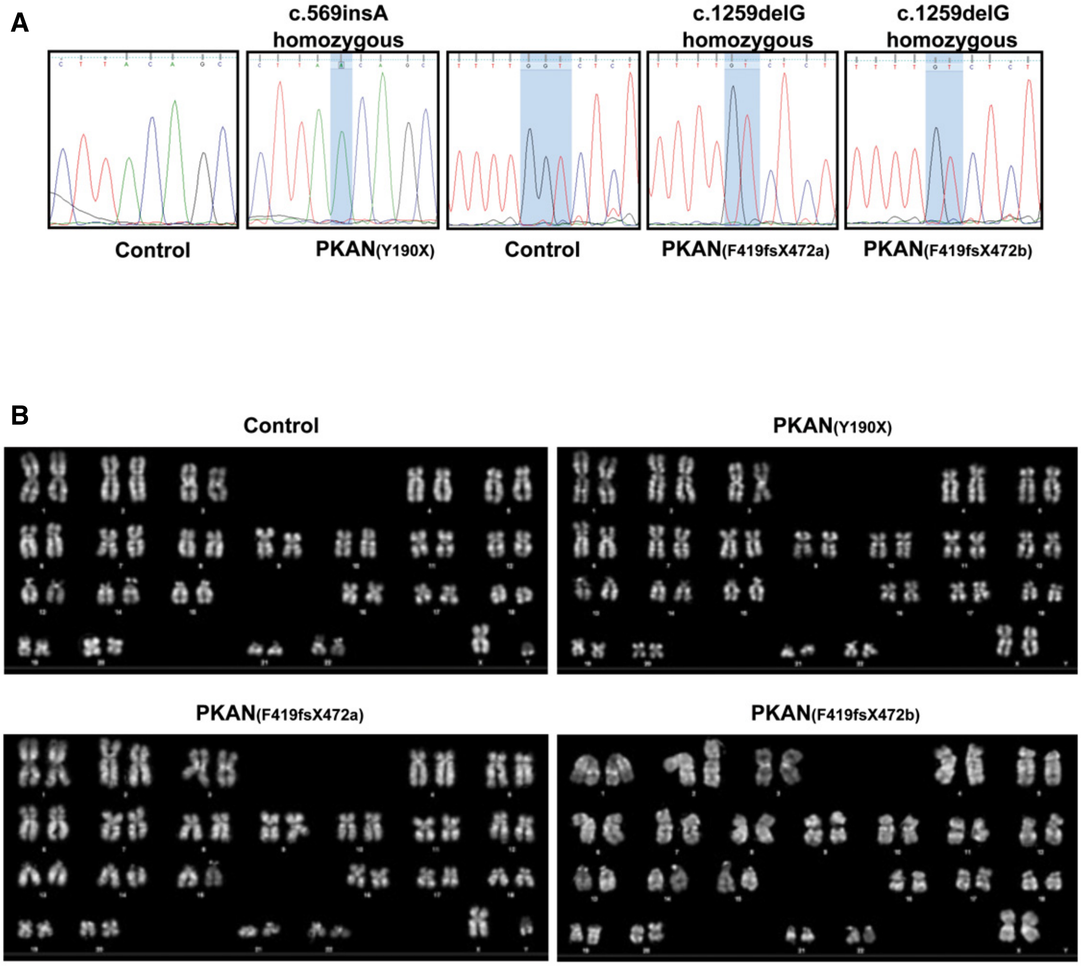

Figure EV2. Characterization of hiPSC clones obtained from fibroblasts of controls and PKAN patients.

A DNA sequence analysis of the hiPSC clones confirmed the presence of *PANK2* mutations.

B Karyotype analysis of hiPSC lines displayed a normal karyotype.

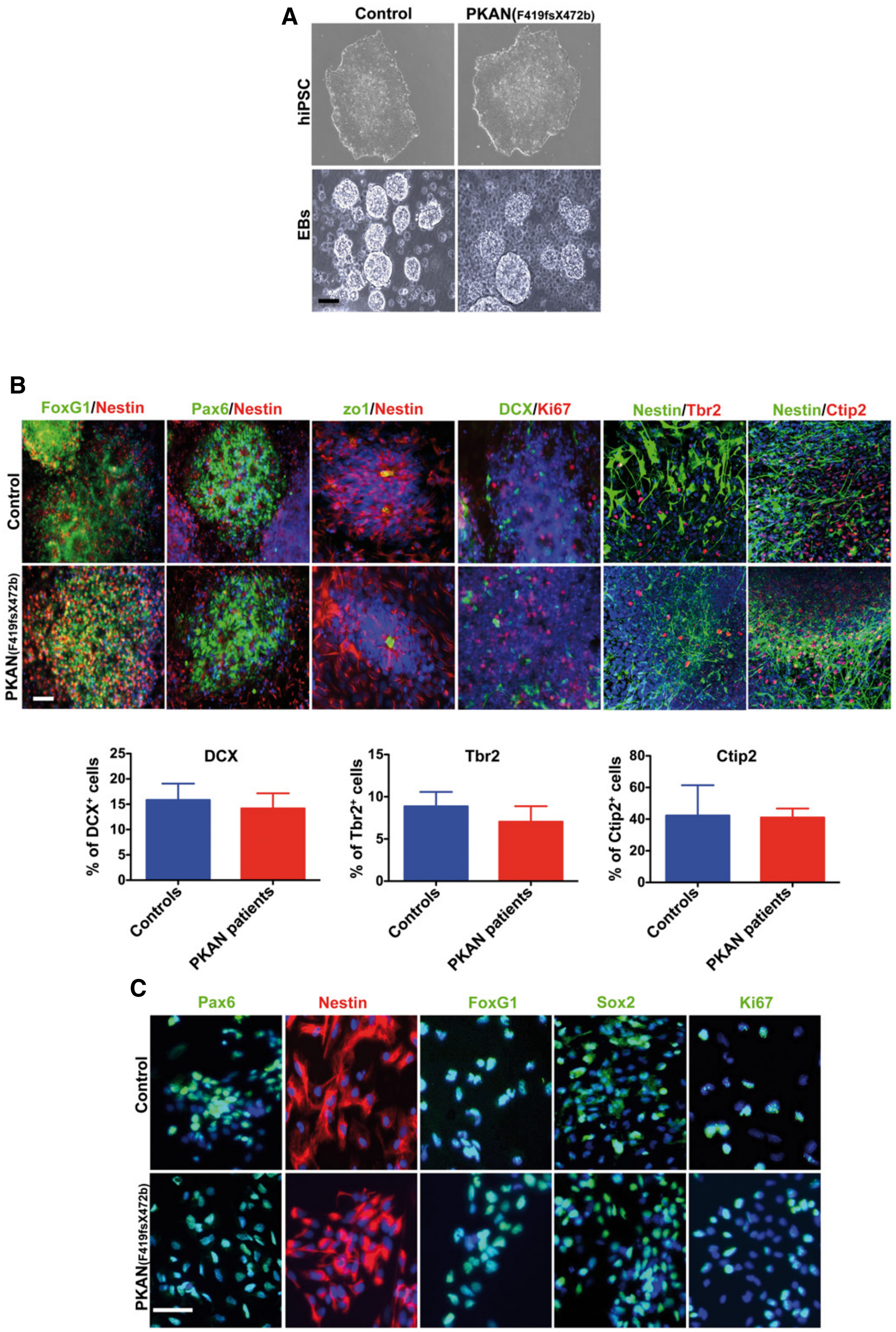

Figure EV3. Generation and differentiation of controls and PKAN patients hiPSC-derived NPCs.

A Representative images of hiPSCs (top) and embryoid bodies (EBs) (bottom) at day 7. Scale bar 100 μm.

B Representative IF images for rosettes at 10 days obtained from EBs. Neural rosette clusters express the typical markers (FoxG1, Nestin, Pax6, zo1, ki67, DCX, Tbr2, Ctip2). Nuclei were stained with Hoechst dye. Scale bar 100 μm. Histograms represent the average percentage + SEM of DCX-, Tbr2- and Ctip2-positive cells of three independent replicates (unpaired two-tailed *t*-test).

C Representative IF images of NPCs obtained from neural rosettes. NPCs expressed the markers Pax6, Nestin, FoxG1, Sox2, and ki67. Nuclei were stained with Hoechst dye. Scale bar 20 μm.

 © 2016 The Authors

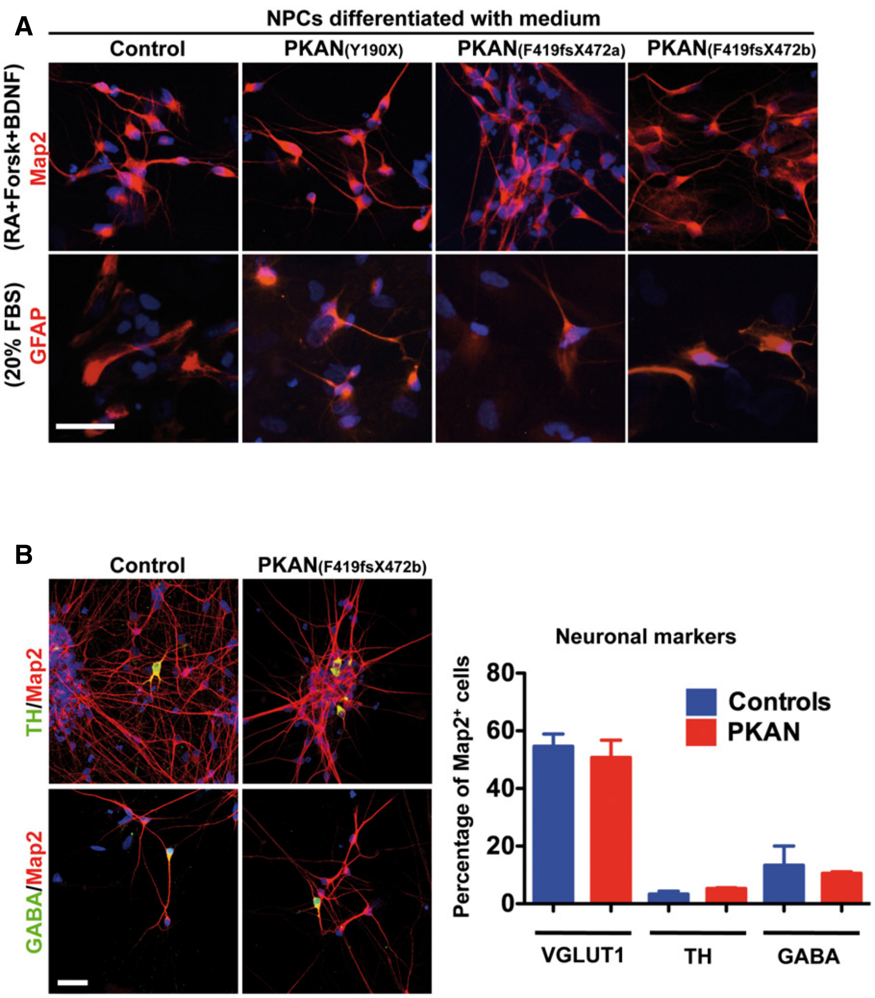

Figure EV4. hiPSC-derived NPCs can be differentiated into neurons (Map2) and astrocytes (GFAP).

A Representative IF images of control and PKAN NPCs differentiated into neurons and astrocytes with specific medium factors. Scale bar 20 μm.

B Representative IF images at 3 weeks of neurons induced with Ngn2 expression from control and PKAN NPCs. Scale bar 20 μm. Nuclei were stained with Hoechst dye. Histogram represents the average percentage of 3 controls and 3 PKAN patients of VGLUT1- (shown in Fig 1B), TH- and GABA-positive neurons, + SEM (unpaired two-tailed *t*-test).
