# Supplementary material for: Coenzyme A corrects pathological defects in human neurons of PANK2‐associated neurodegeneration
Source: EMBO Mol Med. 2016 Aug 11;8(10):1197–211. doi: 10.15252/emmm.201606391 (PMC5048368; doi:10.15252/emmm.201606391)
Supplement: Supplementary file 2 — Table EV1 [file EMMM-8-1197-s002.docx]

**Table EV1.** Oligonucleotides used to clone the PANK2 into Ngn2 lentiviral construct.

| TetO-Ngn2-t2a-Puro modification. | CTAGCGGCAGCGGCGCCACCAACTTCAGCCTGCTGAAGCAGGCCGGCGACGTGGAGGAGAACCCCGGCCCCACCGGTGTTAACT; CTAGAGTTAACACCGGTGGGGCCGGGGTTCTCCTCCACGTCGCCGGCCTGCTTCAGCAGGCTGAAGTTGGTGGCGCCGCTGCCG |
| --- | --- |
| hPANK2 primers | ccgACCGGTATGAGGAGGCTCGGGCCCTTCC; tgcTCTAGAGGCGTAGTCGGGCACGTCGTAG |
