# Supplementary material for: Coenzyme A corrects pathological defects in human neurons of PANK2‐associated neurodegeneration
Source: EMBO Mol Med. 2016 Aug 11;8(10):1197–211. doi: 10.15252/emmm.201606391 (PMC5048368; doi:10.15252/emmm.201606391)
Supplement: Supplementary file 3 — Table EV2 [file EMMM-8-1197-s003.docx]

**Table EV2.** Primers used to characterized iPSC.

| **Gene** | **Forward** | **Reverse** |
| --- | --- | --- |
| KLF4 | TTATTCTCTCCAATTCGCTGACC | GGACTCCCTGCCATAGAGGA |
| SOX2 | CAAGCTCCTTCAACTGGTTCTGT | CTTAGAATGATGCAAGCCAGGTC |
| OCT4 | AGAAAGCGAACCAGTATCGAGAA | CTCAAAATCCTCTCGTTGTGCAT |
| TERT | ACCAAGAAGTTCATCTCCCTGGG | AAAGAAAGACCTGAGCAGCTCGA |
| GDF3 | AAAGGGAACAGTTGACATTGGCC | AGCTACATCCAGCAGGTTGAAGT |
| DPPA2 | GATGCAAAATACCAGCCCTTCCC | CGTTTCCTCGAACATCGCTGTAA |
| FGF4 | CTACAACGCCTACGAGTCCTACA | GTTGCACCAGAAAAGTCAGAGTTG |
| REX1 | CAGATCCTAAACAGCTCGCAGAAT | GCGTACGCAAATTAAAGTCCAGA |
| CMYC | CTGGACACGCTGACGAAAGT | TTCAGCACGCTTCTCCTCCT |
| TDGF | TTTGCTCGTCCATCTCGGG | GCTCCTTACTGTGCTGTATCCC |
| DPPA4 | GACCTCCACAGAGAAGTCGAG | AGGTGGCAGTTTAGAAGGTAATG |
| GADPH | CAAGATCATCAGCAATGCCTCCTG | GCCTGCTTCACCACCTTCTTGA |
| NANOG | CCTCCATGGATCTGCTTATT | ATCTGCTGGAGGCTGAGGTA |
| ACT | ACCCCAGCCATGTACGTT | GGTGAGGATCTTCATGAGGTAG |
| PANK2-exon1 | gggcagaggcatgcacaagt | gcaccaacgagggaccac |
| PANK2-exon4 | ggttcataaatgttaacttcttgttcttat | gatctgcccatctcggc |
