# Supplementary material for: Coenzyme A corrects pathological defects in human neurons of PANK2‐associated neurodegeneration
Source: EMBO Mol Med. 2016 Aug 11;8(10):1197–211. doi: 10.15252/emmm.201606391 (PMC5048368; doi:10.15252/emmm.201606391)
Supplement: Supplementary file 4 — Table EV3 [file EMMM-8-1197-s004.docx]

**Table EV3.** Antibodies used in the study.

| **Antibody-Specie** | **Manufacturer** | **Catalog N°** | **Application** | **Dilution** |
| --- | --- | --- | --- | --- |
| Alexa 488 anti-human CD56 (NCAM) - Ms | BD Biosciences | 557699 | IF | 1:80 |
| Alexa 647 anti-human CD56 (NCAM) - Ms | BD Biosciences | 557711 | IF | 1:80 |
| Oct4 - Rb | Abcam | AB18976 | IF | 1:250 |
| SSEA-1 - Ms | Imm. Sciences | MAB10143 | IF | 1:250 |
| NANOG - Rb | Abcam | AB21603 | IF | 1:250 |
| TRA-1-6 - Ms | Millipore | MAB4350 | IF | 1:250 |
| SOX2 - Rb | Abcam | AB59776 | IF | 1:250 |
| FoxA2 - Rb | Abcam | AB40874 | IF | 1:250 |
| Anti-SMA - Ms | Sigma-Aldrich | A2547 | IF | 1:500 |
| βIII-tubulin/Tuj1 - Ms | Covance | MMS435P100 | IF | 1:500 |
| βIII-tubulin/Tuj1 - Rb | Covance | PRB435P100 | IF | 1:500 |
| FoxG1 - Rb | Abcam | AB18259 | IF | 1:200 |
| Anti human Nestin - Ms | Millipore | MAB5326 | IF | 1:500 |
| Pax6 - Rb | Covance | PRB278P | IF | 1:200 |
| FITC-anti-zo1 | Thermofisher | 33-9111 | IF | 1:200 |
| DCX - Gp | Millipore | AB2253 | IF | 1:200 |
| Ki67 – Rb | Imm. Sciences | AB82549 | IF | 1:200 |
| TH - Rb | Covance | AB10312 | IF | 1:200 |
| GABA – Ms | Sigma-Aldrich | A2052 | IF | 1:200 |
| Map2 – Ms | Imm. Sciences | MAB10334 | IF | 1:400 |
| Tbr2 - Rb | Abcam | AB23345 | IF | 1:200 |
| Ctip2-Rat | Abcam | AB18465 | IF | 1:200 |
| Map2 – Rb | Imm. Sciences | AB5622 | IF | 1:400 |
| GFAP - Ms | Millipore | MAB360 | IF | 1:200 |
| hNu – Ms | Millipore | MAB1281 | IF | 1:200 |
| NeuN – Ms | Millipore | MAB377 | IF | 1:200 |
| Pank2 clone 3H9 - Ms | Origene | TA501321 | IF/WB | 1:200/1:3000 |
| PanNav - Ms | Sigma-Aldrich | S8809 | IF | 1:100 |
| Vglut1 - Gp | Synaptic System | 135304 | IF | 1:200 |
| mAco – Rb | Antibody Verify | AAS90403C | WB | 1μg/ml |
| cAco - Rb | Home made | Campanella etal, 2012 | WB | 1:500 |
| TfR1 - Ms | Zymed laboratories | 13-6800 | WB | 1:2000 |
| FtH | Home made | Luzzago etal, 1986 | WB | 1:2000 |
| Actin - Ms | Sigma-Aldrich | A5441 | WB | 1:6000 |
| Anti-mouse HRP | Sigma-Aldrich | AP130P | WB | 1:100000 |
| Anti-rabbit HRP | Sigma-Aldrich | AP156P | WB | 1:50000 |
| Anti-mouse-546 | Imm. Sciences | IS20305 | IF | 1:800 |
| Anti-rabbit-488 | Imm. Sciences | IS20014 | IF | 1:800 |
| Anti-guinea pig-594 | Mol. Probes | A11076 | IF | 1:800 |
